# Radiation-induced tumor following pencil beam scanning proton therapy for pediatric intracranial ependymoma: a pragmatic analysis of risk factors

**DOI:** 10.1093/jrr/rrag066

**Published:** 2026-08-20

**Authors:** Eymeric Le Reun, Arturs Meijers, Maria Teresa Romero-Expósito, Ilya Kotov, Daniel J Indelicato, Damien Charles Weber

**Affiliations:** Center for Proton Therapy, Paul Scherrer Institute, Forschungsstrasse 111, 5232 Villigen PSI, Switzerland; Center for Proton Therapy, Paul Scherrer Institute, Forschungsstrasse 111, 5232 Villigen PSI, Switzerland; The Skandion Clinic, von Kraemers Allé 26, 752 37, Uppsala, Sweden; Department of Oncology-Pathology at Karolinska Institutet, Anna Steckséns gata 30A, D2:04, 171 64 Solna, Sweden; Center for Proton Therapy, Paul Scherrer Institute, Forschungsstrasse 111, 5232 Villigen PSI, Switzerland; Department of Radiation Oncology, University of Florida College of Medicine, 653-1 West 8th Street, Jacksonville, FL 32209, USA; Center for Proton Therapy, Paul Scherrer Institute, Forschungsstrasse 111, 5232 Villigen PSI, Switzerland; Department of Radiation Oncology, Inselspital, Bern University Hospital, University of Bern, Freiburgstrasse 18, 3010 Berne, Switzerland

**Keywords:** secondary tumor, radiation-induced tumors, proton therapy, pencil beam scanning, intracranial ependymoma, children, pediatric oncology

## Abstract

Radiation-induced tumors (RITs) represent a rare yet serious long-term complication of ionizing radiation, particularly relevant in pediatric patients due to their long life expectancy. Proton therapy (PT) is widely used in children because of its favorable dose distribution compared to photon therapy. However, PT generates secondary neutrons that may contribute to RIT development. In a previously reported cohort of 119 children treated with pencil beam scanning (PBS) PT for intracranial ependymoma at the Paul Scherrer Institute (PSI), four RITs (3.4%) were observed after a median follow-up of 5.3 years. The present study aimed to explore potential risk factors for RITs within our institution’s PBS-PT treatment protocol. A literature review identified one comparable cohort of 51 pediatric patients treated with commercial-like PBS-PT systems, in which no RITs were reported after a median follow-up of 5 years. In parallel, Monte Carlo simulations were performed to compare neutron production between the two PSI gantries. Neutron dose calculations revealed higher secondary neutron exposure associated with Gantry 1 compared to Gantry 2. Variability in system-specific neutron dose emissions may contribute to differential RIT risks. However, given the limited number of events, differences in follow-up duration, and potential confounding factors such as chemotherapy and treatment volumes, these findings should be considered hypothesis-generating. Continued optimization of PT delivery and long-term monitoring of pediatric patients treated with PBS-PT are required to establish causal relationship.

## INTRODUCTION

In pediatric radiation oncology, particular attention is paid to radiation-induced secondary events, as these patients have a long-life expectancy [1, 2]. From this point of view, proton therapy (PT) has produced equal or better results than conventional photon radiotherapy (RT) [3–7]. However, clinical outcomes have shown no significant radiation-induced tumor (RIT) rate difference between PT and RT in children [8].

During PT, interactions between the primary proton beam and either treatment beamline components or patient tissue can result in the production of secondary particles, including protons, electrons, alpha particles and neutrons [9]. Among these, neutrons are of particular concern due to their high relative biological effectiveness and potential contribution to RITs [10–12].

It is noteworthy that PT can be delivered according to two main different delivery techniques. While passive-scattering (PS) has been the most commonly PT technique since the 1950s [13], pencil beam scanning (PBS), or spot-scanning, was first introduced in the late 1990s at the Paul Scherrer Institute (PSI) for the routine treatment of cancer patients and is currently the main delivery technique worldwide [14].

In a previously published series of 119 children treated with PBS-PT for intracranial ependymoma (*n* = 119), in which more than two-thirds (69.7%) of the PSI cohort patients received PBS-PT using the Gantry 1 in-house system, secondary brain tumors occurred in 4 cases (3.4%), after a median follow-up (FU) of 5.3 years (range: 0.1–18.2) and a median latency to diagnosis of 9.3 years (range: 3.7–15) [15]. Each of the four secondary tumors had a distinct histology from that of the primary ependymoma (meningioma, astrocytoma, glioblastoma), which is a criterion for classifying them as RIT (Table 1). In details, three of these four cases occurred among the 83 patients treated using the PSI in-house Gantry 1 system (3.6%), and one case among the 34 patients treated with Gantry 2 (2.9%) which is also built in-house, but with regards to range shifter management resembles commercial-like systems (Fig. 1 and Table 1).

**Table 1:** Radiation-induced tumors in children treated for intracranial ependymoma with PBS-PT at PSI.

Case	PSI Gantry	Chemotherapy	GTV to CTV margin (cm^3^)	PTV (cm^3^)	RIT isodose (range) [Gy RBE], region	Age at PT (months)	Time from PT to RIT (months)	RIT histology	FU time (months)	Status at last FU
1	Gantry 1	HIT 2000	10	193.2	30 (17–50), posterior fossa	27.7	180.5	Meningioma, WHO grade 1	185.5	Alive without disease
2	Gantry 1	Carboplatin–Etoposide–Ifosfamide	10	141.6	59.4 (50–59.4), posterior fossa	28.8	123.8	Glioblastoma	142.6	Dead from RIT
3	Gantry 1	Irinotecan–Cisplatin	10	145.0	40 (20–55), posterior fossa	14.7	100.0	Glioblastoma	102.2	Dead from RIT
4	Gantry 2	Irinotecan–Cisplatin	5	211.8	20 (10–58), periventricular region	166.7	44.8	Astrocytoma, WHO grade 3	50.2	Dead from primary tumor

PBS = pencil beam scanning, PT = proton therapy, PSI = Paul Scherrer Institute (Switzerland), RBE = relative biological effectiveness, WHO = World Health Organization, RBE = relative biological effectiveness, GTV = gross tumor volume, CTV = clinical target volume, PTV = planning target volume, RIT = radiation-induced tumor.

**Fig. 1:**
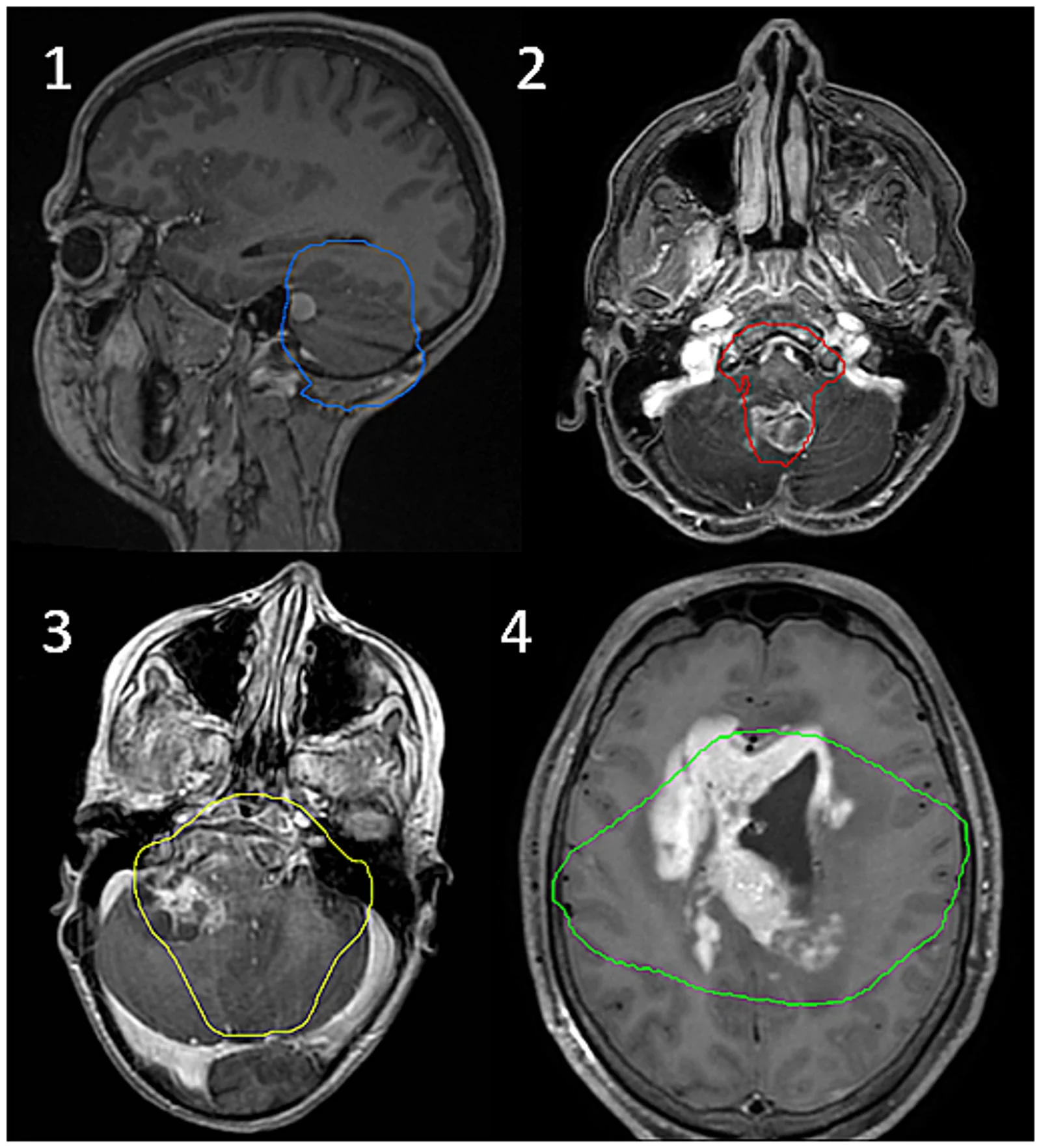
Radiation-induced tumors after pencil beam scanning proton therapy (PBS-PT) at PSI. Case 1: secondary meningioma WHO grade 1 (infra-tentorial T1 hypersignal), with 30 Gy isodose line (blue). Case 2: secondary WHO grade 4 gliobastoma (infra-tentorial T1 hypersignal), with 59.4 Gy isodose line (red). Case 3: secondary WHO grade 4 gliobastoma (infra-tentorial T1 hypersignal), with 40 Gy isodose line (yellow). Case 4: secondary anaplastic WHO grade 3 astrocytoma (periventricular T1 hypersignal), with 20 Gy isodose line (green).

Given this relatively high rate of RITs in our institution and the potential involvement of proton-induced neutrons, this study was intended as an exploratory and hypothesis-generating analysis, aiming to identify potential risk factors for RITs within our institution’s PBS-PT treatment protocol.

## MATERIALS AND METHODS

### Literature review

We performed a literature review of all series of pediatric intracranial ependymoma treated with PT. Series including adult patients (>21 years-old), and/or extracranial tumors, and/or non-ependymomas, and/or re-irradiation were excluded. In case of missing data, a request was sent electronically to collect more detailed information. For frequency analysis, we used Fisher’s exact test for two-way tables [16].

### Analysis of the proton-induced neutron dose

To evaluate the difference in neutron production between PSI in-house PBS devices Gantry 1 and Gantry 2, a straightforward simulation was conducted using the Monte Carlo N-Particle code [17]. The following three irradiation scenarios, delivering the same dose to a point at a depth of 4 cm in water, were considered:

- A 70 MeV pristine proton beam directed at the water phantom (‘no range shifter’ scenario).
- A 177 MeV pristine proton beam directed at the water phantom, passing through 16.08 cm polystyrene range shifter (‘Gantry 1 scenario’).
- A 98 MeV pristine proton beam directed at the water phantom, passing through 3.062 cm lexan range shifter (‘Gantry 2 scenario’)

## RESULTS

### Cohorts selection

A recent systematic literature review, published in 2024, assessed publications of young patients with brain tumors treated with PT [18]. Among the five articles reporting outcomes of children with intracranial ependymoma [19–23], we excluded one dealing with re-irradiation [20], another published by our PSI team in 2016 [19] and two [21, 22] reporting series included in a more recent publication from Indelicato *et al.* [24]. At the end, only the Sato *et al.* publication [23] was selected among the five articles of this review. In addition, two other articles were identified reporting clinical outcomes of older cohorts, but with a median FU of more than 5 years [25, 26]. We identified another recent article [27], but this cohort was also included in the Indelicato *et al.* publication [24]. Furthermore, we have requested more detailed data regarding three series [23, 25, 26], but unfortunately did not get any response.

Ultimately, the screening process and paper selection shortlisted only the Indelicato *et al*. clinical series [24], in addition to our PSI series [15], as detailed in Fig. 2.

**Fig. 2:**
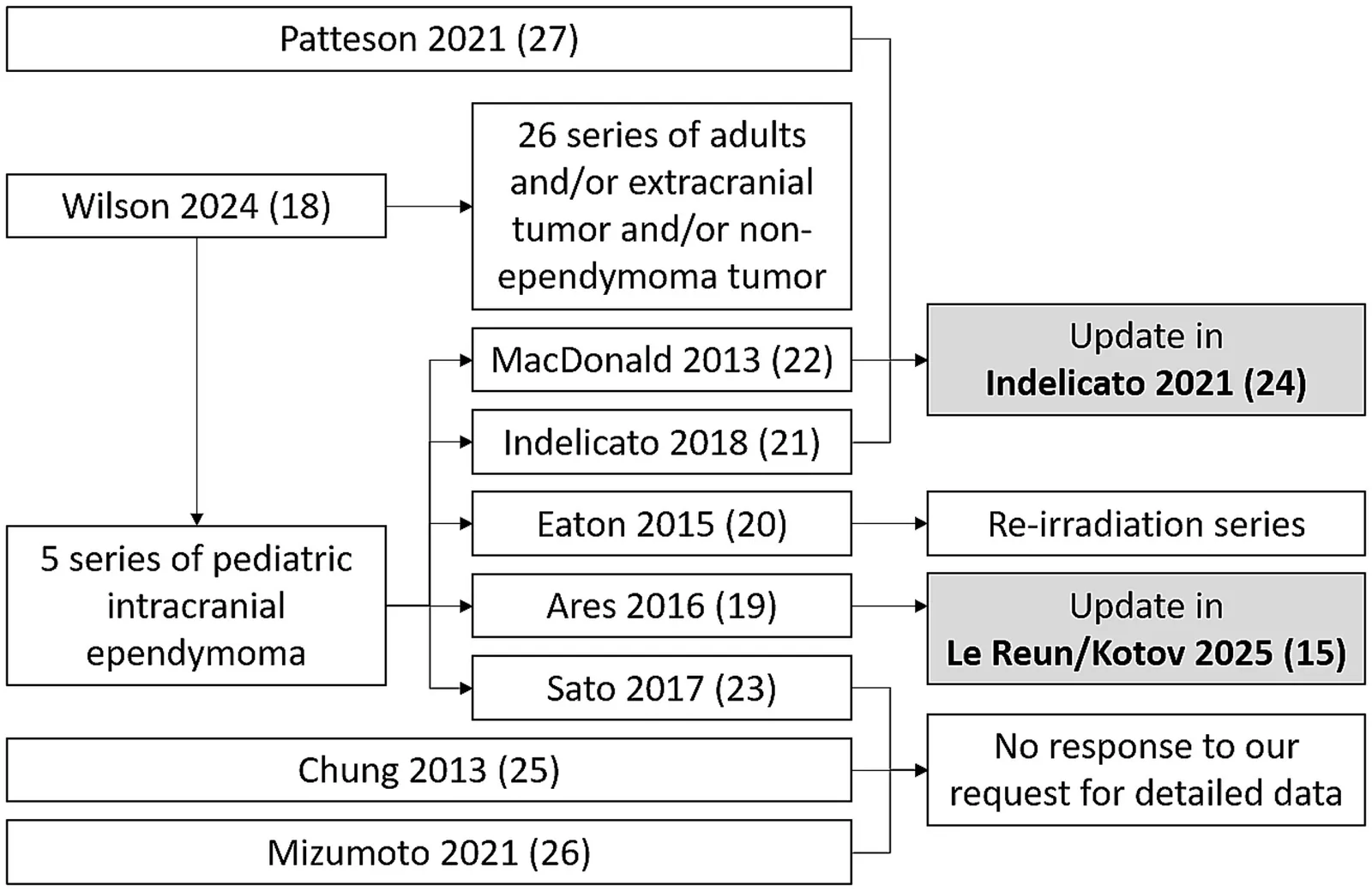
Study flow chart. The selection process highlighted only two articles (highlighted in gray fill) dealing with updated data on pediatric intracranial ependymoma series.

### Description of the US-cohort

Indelicato *et al*. reported the outcomes of 386 ependymoma patients treated with PT, either at Massachusetts General Hospital (MGH, Boston, MA, USA) or University of Florida Health Proton Therapy Institute (Jacksonville, FL, USA), between 2000 and 2019 [24] (Table 2). Median age at PT was 3.8 years (0.7–21.3). Nearly two-thirds of the cohort had anaplastic ependymoma (64.5%), and most tumors were infra-tentorial (71.0%). All patients underwent surgery, chemotherapy was used in 27.2% of cases, PT was delivered by PBS in 13.2% of the cases (*n* = 51), and mostly under anesthesia (66.8%). The median prescribed dose was 55.8 Gy_RBE_ (50.4–59.4) in 1.8 Gy_RBE_ per fraction. Median FU was 5.0 years (0.4–16.7). The 5 years local control, progression-free survival and overall survival were respectively 79.2% (74.4–83.3), 68.4% (63.3–73.1) and 85.2% (80.7–88.9). Two patients died from grade 5 toxicity (0.5%). A total of 5 patients (1.3%) developed RIT, at a median time of 8 years (range: 4–14) after PT, and all of them after PS-PT. No RIT were reported in the PBS sub-cohort, where patients were treated with a commercial-like PBS system (Table 2).

**Table 2:** Outcomes of proton therapy in pediatric intracranial ependymoma

	Le Reun *et al.*[15]	Indelicato *et al.*[24]	Statistical estimation: Mann–Whitney^*^ Chi-2^#^ Fisher^¤^
**Number of patients**	*n* = 119	*n* = 386	
**Treatment period** (years)	2004–2022	2000–2019	N/A
**Age at proton therapy:** median (range) (years)	3.0 (0.8–17.8)	3.8 (0.7–21.3)	*P* ≈ 0.30^*^
**Median time of follow-up:** median (range) (years)	5.3 (0.1–18.2)	5.0 (0.4–16.7)	*P* ≈ > 0.05^*^
**Gender**			
Male	77 (64.7%)	216 (56.0%)	*P* = 0.10^#^
Female	42 (35.3%)	170 (44.0%)	
**Tumor grade**			
Classic (WHO grade 2)	20 (16.8%)	137 (35.5%)	***P* < 0.001** ^#^
Anaplastic (WHO grade 3)	98 (82.4%)	249 (64.5%)	***P* < 0.001** ^#^
Ependymoblastoma (WHO grade 4)	1 (0.8%)	0	*P* ≈ 1^¤^
**Tumor location**			
Supra-tentorial	35 (29.4%)	112 (29.0%)	*P* = 0.92^#^
Infra-tentorial	84 (70.6%)	274 (71.0%)	*P* = 0.92^#^
**Chemotherapy**			
Yes	73 (61.4%)	105 (27.2%)	***P* < 0.001** ^#^
No	46 (38.6%)	281 (72.8%)	
**Proton therapy prescription**			
Proton therapy technique			
Passive scattering^*^	0	335 (86.8%)	***P* < 0.001** ^¤^
Pencil beam scanning^*^	119 (100%)	51 (13.2%)	***P* < 0.001** ^¤^
*PSI Gantry 1*	*83/119 (69.7%)*	*N/A*	*N/A*
*PSI Gantry 2*	*34/119 (28.6%)*	*N/A*	*N/A*
*PSI Gantry 3*	*2/119 (1.7%)*	*N/A*	*N/A*
General anesthesia			
Yes^*^	102 (85.7%)	258 (66.8%)	***P* < 0.001** ^#^
No^*^	17 (14.3%)	128 (33.2%)	
Total prescribed dose: median (range) [Gy, RBE]	59.4 (44.0–63.4)	55.8 (50.4–59.4)	N/A
**Proton therapy target volumes**			
GTV to CTV margin (range) (mm)	10 (4–20)	N/A	N/A
PTV (range) (cm^3^)	131.0 (42.3–407.5)	N/A	N/A
**Disease control**			
5-years local control: median (range)	70.4% (62.2–79.7)	79.2% (74.4–83.3)	N/A
5-years progression-free survival: median (range)	63.5% (55–73.3)	68.4% (63.3–73.1)	N/A
5-years overall survival: median (range)	82.2% (75–90.1)	85.2% (80.7–88.9)	N/A
**Fatal grade 5 toxicity**	1 (0.8%)	2 (0.5%)	*P* ≈ 1^¤^
**Radiation-induced tumors**			
Passive scattering^*^	*N/A*	5 (1.3%)	
Pencil beam scanning^*^	4 (3.4%)	*0*	*P* = 0.18^¤^
*PSI Gantry 1*	3/83 (3.6%)	*N/A*	*N/A*
*PSI Gantry 2*	1/34 (2.9%)	*N/A*	*N/A*
*PSI Gantry 3*	0	*N/A*	*N/A*
Time to RIT: median (range) (years)	9.3 (3.7–15)	8.0 (4.0–14)	*P* > 0.05^*^

RBE, relative biological effectiveness; PT, proton therapy; No. pts, number of patients; WHO, World Health Organization; GTV, gross tumor volume; CTV, clinical target volume; PTV, planning target volume; PSI, Paul Scherrer Institute (Switzerland); N/A, not applicable; RIT, radiation-induced tumor.

### Proton-induced neutron dose according to PBS gantry at PSI

The fact that patients treated with PBS-PT in Indelicato’s series did not experience any RIT suggests that the high rate of RITs in our cohort might stem from a cause inherent to PSI. Consequently, the technical characteristics of PSI’s Gantry 1 warrant particular attention, as most patients in the PSI cohort received treatment using this in-house PBS system.

Figure 3 shows the ratio of neutron dose equivalent for the range shifter scenarios relative to the no range shifter scenario at the isocenter plane at PSI. It can be observed that the PSI PBS-Gantry 1 scenario results in a considerably higher neutron production around the proton beam compared to PBS-Gantry 2. For example, in the region immediately after the Bragg peak, the increase is about a factor of 20 for Gantry 1, whereas for Gantry 2 it is only about a factor of 6. In absolute terms, the dose equivalent both in-field and around the proton beam ranged from 0.01 to 0.14 mSv/Gy, which can be considered relatively low.

**Fig. 3:**
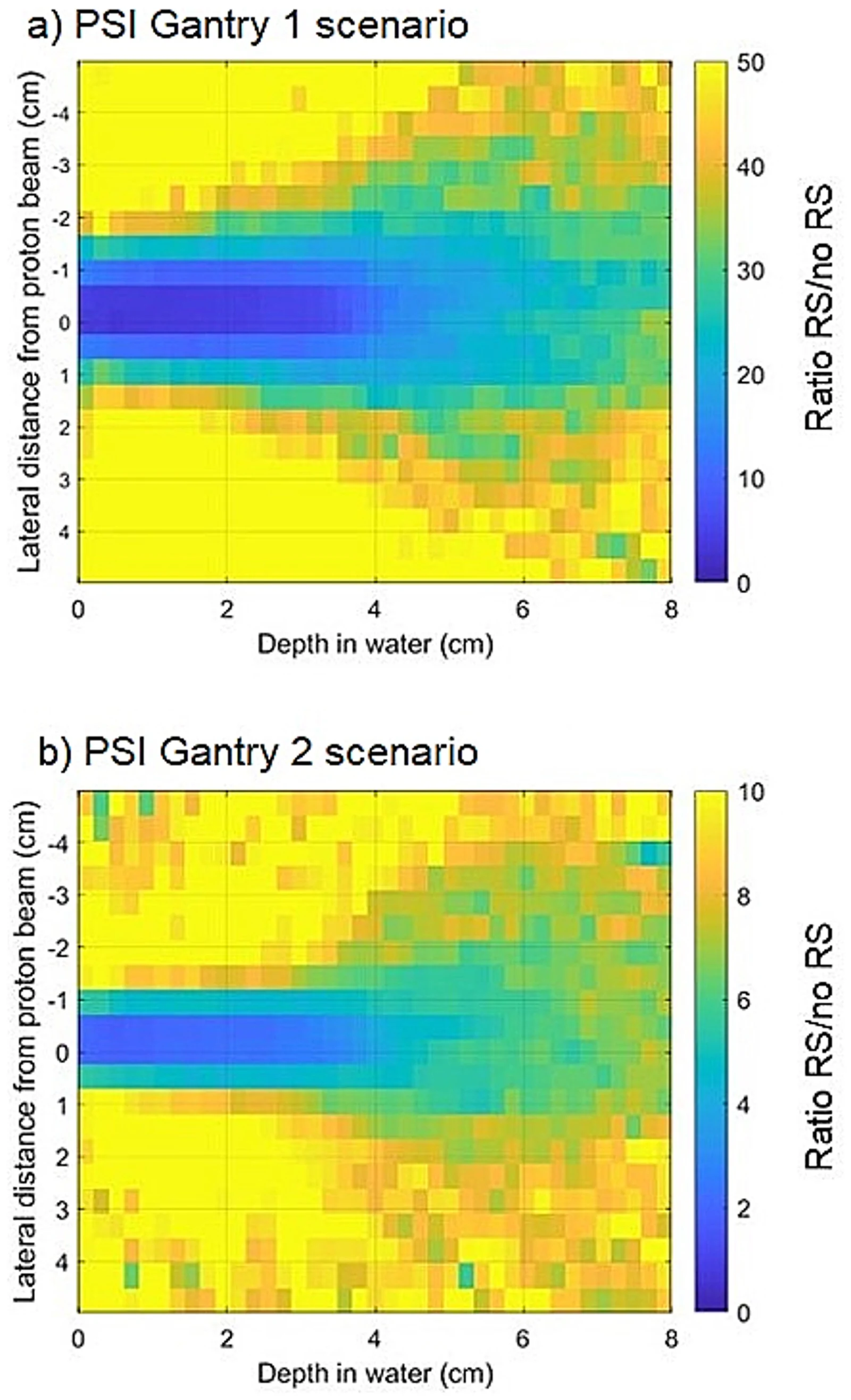
Ratio of neutron dose equivalent per absorbed dose for the range shifter (RS) scenarios—PSI Gantry 1 (a) and Gantry 2 (b)—relative to the no RS scenario. The maps were obtained around pristine Bragg peak at isocenter plane. Note that a different scale is used for each plot.

## DISCUSSION

We analyzed a total of 505 pediatric patients treated with PT for intracranial ependymoma, including 119 patients from the PSI [15] and 386 patients from the UF/MGH cohort [24] (Table 2). While Indelicato *et al*. reported no RIT in their PBS sub-cohort of 51 patients, our PSI PBS-cohort exhibited four cases of RIT (3.4%). Given the very limited number of RIT events, and the fact that the two cohorts are not fully comparable, no meaningful statistical comparison between RIT risk factors could be performed. However, several etiological hypotheses can be proposed.

### Individual factors

The development of RITs may be modulated by a range of risk factors. Among them, younger children under 3 are more susceptible to secondary tumors due to ongoing tissue development and sensitivity to radiation [28–31]. Individuals with genetic syndromes, such as Neurofibromatosis type 2—which is also associated with ependymoma [32]—may also have an increased risk of developing secondary tumors due to inherent susceptibility. It is noteworthy that no genetic syndromes were reported in our PSI cohort, although genetic syndrome were not actively diagnosed and were passively registered, as in the US cohort. As such, we cannot exclude under-reporting and an imbalance of genetic conditions in disfavor of one cohort.

### Prescribed treatment

#### Use of chemotherapy

Chemotherapy agents are well-known carcinogens [33, 34]. In addition, it must be stressed that the combination of chemotherapy and radiation may further increase the risk of RIT [8]. In fact, all four patients who developed RIT in our PSI cohort also underwent adjuvant chemotherapy. Of note, more children were treated with chemotherapy in the PSI group (61%) than in the US cohort (27%; *P* < 0.001; Table 1). This significant difference could explain the disparity in RIT observed between the two PBS cohorts.

#### Proton therapy volume

According to Diallo *et al*. [35], most RITs might occur at the periphery of the treatment field, often in areas receiving doses below 2.5 Gy. As a result, larger treatment volumes would increase exposure of normal tissue to radiation, raising the risk of secondary tumors.

In the PSI series, PTV volumes of patients with RIT were 142–212 cm^3^ (median: 169 cm^3^) (Table 1), which is above-average volume size of 131 cm^3^ (range: 42.3–407.5) in the cohort (Table 2), especially if patient age is taken into consideration. Difference in PT volumes may significantly influence RIT risk.

#### Proton dose

The higher the radiation dose prescribed, the greater the likelihood of RIT appearing in the surrounding tissues. Although RBE varies along the proton beam path with increasing LET, a proton RBE of 1.1 has traditionally been used in clinical practice and for dose reporting in this study. LET typically ranges from under 2 keV/μm at entry to over 10 keV/μm at the distal fall-off, leading to increased RBE. In our series, RITs were not consistently observed in regions exposed to the distal edge of the beam. Three out of the four patients who developed RIT were treated with a prescribed dose of 59.4 Gy_RBE_ (range within the cohort 44.0–63.4 Gy_RBE_). In the PSI cohort, all RITs arose within isodose regions of at least 10 GyRBE, with some cases occurring directly within the PTV (Fig. 1). These findings challenge the classical low-dose-induced RIT paradigm and suggest that total dose alone cannot account for the occurrence of RITs in the PSI cohort.

### Potential role of proton-induced neutrons from Gantry 1 in the development of RITs

In our PBS cohort, most RIT cases (3 of 4) occurred in patients treated with the in-house Gantry 1 system (Table 1). However, given the very limited number of events, no statistically significant difference in RIT incidence can be demonstrated between Gantry 1 and Gantry 2.

Unlike most contemporary commercial PBS systems—similarly as PSI’s own Gantry 2—which employ an energy selection system (ESS) located outside the treatment room to modulate beam energy, Gantry 1 relies on an internal mechanism in the nozzle for energy modulation. Specifically, Gantry 1 utilizes a set of 40 range shifter plates positioned at the exit of the treatment nozzle to adjust the proton beam energy [14]. Only three discrete proton beam energies (up to a maximum of 177 MeV) could be delivered to the treatment room, while energy modulation was achieved by varying the number of range shifter plates in the nozzle.

As a result, the total water equivalent thickness (WET) of the range shifter in Gantry 1 reached approximately 18 cm—substantially higher than the typical 3–7 cm WET range shifters employed in commercial systems. This design likely contributed to increased neutron production in close proximity to the patient, which may have partially contributed to the higher neutron dose in patients treated with Gantry 1.

In Fig. 3, it can be observed that the PSI Gantry 1 scenario results in a considerably higher neutron production around the proton beam compared to Gantry 2. If the ‘no range shifter’ scenario is taken as representative of a pure PBS facility, the resulting ratio is comparable to values reported when comparing PBS and PS facilities [36]. However, since neutron production also depends on the target size, a direct comparison with actual clinical treatment plans is not straightforward.

Nevertheless, in absolute terms, the dose equivalent both in-field and around the proton beam ranged from 0.01 to 0.14 mSv/Gy, which can be considered relatively low, and thus arguable if that could induce secondary tumor. Indeed, the ANDANTE project, studying the risk of RIT from neutron exposure, indicated that it could take decades to determine the carcinogenic effects of PT-related neutron exposure through epidemiological FU [37].

Furthermore, because this neutron dose analysis is purely dosimetric and not patient-specific, no causal relationship can be formally established.

### Follow-up

Given the low incidence of RITs and the prolonged and highly variable latency period, patients who are lost to FU can substantially affect the recorded and reported rates of secondary tumor occurrence. According to Cahan *et al.* [38], a secondary tumor may be considered radiation-induced if it arises after a latency period of at least two years following irradiation. However, clinical outcomes showed that this latency can range from around 5 years (range, 2–30 years) [39] and up to 14.6 years (range, 2–33 years) [40].

In this regard, although the FU periods were comparable between the two groups (5.3 years at PSI versus 5.0 years in the US cohort), they remained short in relation to the length of FU required to detect the onset of RITs. Consequently, the absence of RITs in the US PBS-cohort may reflect insufficient latency rather than true absence of risk.

This long-term observational study identified a notably high incidence of radiation-induced tumors (RITs) among pediatric patients with intracranial ependymoma treated with PBS-based proton therapy (PBS-PT) at our institution compared with other reported PBS cohorts, although direct comparisons remain limited by differences in patient characteristics, treatment approaches and follow-up duration.

Variability in system-specific neutron dose emissions may contribute to differential RIT risks. However, given the limited number of events, differences in follow-up duration and potential confounding factors such as chemotherapy and treatment volumes, these findings should be interpreted with caution. Continued optimization of PT delivery and long-term monitoring of pediatric patients treated with PBS-PT are required to establish a causal relationship.
